# Occult pulmonary arterial hypertension in patients with previous pulmonary tuberculosis

**DOI:** 10.7196/AJTCCM.2020.v26i4.110

**Published:** 2020-12-01

**Authors:** I S Kalla, A Miri, F Seedat

**Affiliations:** Division of Pulmonology, Department of Internal Medicine, School of Clinical Medicine, Faculty of Health Sciences, University of the Witwatersrand, Johannesburg, South Africa

**Keywords:** Pulmonary tuberculosis, Pulmonary arterial hypertension

## Abstract

**Background:**

Pulmonary tuberculosis (TB) still causes a significant public healthcare burden. Despite successful treatment, TB can lead to permanent lung damage and pulmonary hypertension (PH). PH can also occur in the absence of significant lung damage, leading clinicians to question whether pulmonary TB may cause pulmonary arterial hypertension (PAH), an entity that has not been otherwise described.

**Objectives:**

To determine the prevalence of PAH in patients previously treated for TB.

**Methods:**

We recruited 20 participants who were previously treated for TB and had no other underlying risk factors for the development of PH. The participants underwent electrocardiography (ECG), chest radiography, lung function tests and echocardiography (ECHO). Data from these non-invasive investigations were evaluated to determine findings that were suggestive of PH.

**Results:**

At a median duration of 30 months from diagnosis of TB, no participant had echocardiography findings that were suggestive of PH (pulmonary artery pressure (PAP) ≥40 mmHg). However, there was a negative correlation between the time from diagnosis and right ventricular dysfunction assessed by measuring a tricuspid annular plane systolic excursion (r=–0.5136; p=0.0205). Furthermore, one-third of the participants (n=7) had one or more ECG features supporting PH and 85% of the participants (n=17) demonstrated at least one chest X-ray (CXR) feature of PH.

**Conclusion:**

Although our study did not demonstrate ECHO findings supporting PH, ECG and CXR modalities were suggestive. Therefore, future studies consisting of larger cohorts and including the use of other sensitive modalities such as computed tomography are warranted. Moreover, these studies will need to determine whether the entity of PAH secondary to previously treated pulmonary TB exists.

## Background

Tuberculosis (TB) is ranked amongst the top 10 leading causes of death worldwide and is the leading cause of death from a single infectious agent.^[1]^ South Africa (SA) has a high burden of TB, with ~438 000 new cases reported in 2019.^[2]^ The Department of Health in SA has implemented strategies that have been successful in curbing the incidence of TB.^[3]^

Despite successful treatment, TB may cause significant long-term cardiorespiratory complications that are well known, including fibrocavitary changes, bronchiectasis, chronic pulmonary aspergillosis and chronic obstructive pulmonary disease.^[4]^ These complications may have further sequelae such as the development of pulmonary hypertension (PH) and right heart failure, which substantially impacts quality of life and further burdens the healthcare system.^[5]^

Pulmonary hypertension is defined as an increase in mean pulmonary artery pressure (PAP) ≥20 mmHg at rest as assessed by right heart catheterisation (RHC) as gold standard.^[6,7]^ Other modalities such as echocardiography may be used to determine the probability of PH.^[6]^ This disease is associated with significant morbidity and mortality.^[8]^ Therefore, it is crucial that those suspected to be at risk of disease are identified and be put on treatment as early as possible.

An under-recognised cause of PH associated with previously treated TB is the development of a vasculitis of the pulmonary artery.^[9]^ This may lead to an increase in pulmonary arterial vascular resistance and subsequently PH.^[10]^ Other mechanisms that have been described to lead to the development of PH include obliterative changes of the pulmonary arteries and an endarteritis obliterans in the vessels following TB,^[11]^ similar to those noted in the development of pulmonary arterial hypertension (PAH).

Specific targeted therapy has recently been developed for PAH based on its underlying pathophysiology. These include endothelin receptor antagonists, phosphodiesterase type 5 inhibitors, prostacyclin analogues and prostacyclin receptor agonists.^[6]^ Early and appropriate administration of these drugs has been shown to be efficacious in alleviating symptoms, improving the haemodynamic profile and delaying the time to worsening of clinical symptoms.^[12]^ Many of these therapies are not currently available in the SA public healthcare sector due to their costs.

Previous studies have demonstrated that PAH can develop in patients with minimal lung complications in countries with a high burden of TB.^[13,14]^ In fact, Allwood *et al*.^[14]^ noted that significant destruction of the pulmonary vasculature, in the absence of extensive parenchymal disease following TB, is an unexplored entity and may result in post-TB associated PAH.^[14]^ They suggested that this paucity in the literature underscores a need for future studies to resolve this challenge. The outcomes of these studies will have serious implications on the burden of TB disease, the need for early detection of PAH and the need to dispense appropriate treatment in order to minimise disease morbidity. The aim of this study was to investigate whether PAH was present in participants who were previously treated for TB.

## Methods

Participants who were previously treated for TB were recruited and enrolled into this pilot prospective cohort study between October 2018 and April 2019. The study was undertaken in the Department of Infectious Diseases at Charlotte Maxeke Johannesburg Academic Hospital (CMJAH), a quaternary hospital in Gauteng Province, SA. Participants were identified from the TB registry kept in the Outpatient Department or from those who attended follow-up visits after completing their TB treatment.

Participants with active TB and those with known aetiologies of PH such as HIV, chronic obstructive pulmonary disease, interstitial lung disease, obstructive sleep apnoea, autoimmune diseases, connective tissue disorders, collagen vascular disorders, liver cirrhosis, chronic bilharzia, heart disease and current or previous pulmonary embolus or deep vein thrombosis were excluded from the study. A chest X-ray (CXR) was subsequently performed to assess the presence of parenchymal lung disease. Participants who had severe bronchiectasis were risk stratified using the FACED score developed by Martinez-Garcia *et al*.^[15]^ The score uses the forced expiratory volume in one second (FEV1), age, chronic microbial colonisation, radiological extent and dyspnoea to stratify patients with non-cystic bronchiectasis. Patients with a FACED score ≥5 were excluded from the study.

A structured questionnaire was used to record demographics, details of TB diagnosis (date, method and duration of therapy) and TB drug sensitivity. Each participant underwent an electrocardiogram (ECG), pulmonary function test (spirometry and diffusing capacity of lung for carbon monoxide (DLCO)), echocardiography (ECHO) and CXR to determine features that were indicative of PAH. The data were interpreted by a pulmonologist in the Division of Pulmonology at CMJAH. A diagnosis of PH was made based on a peak systolic pulmonary artery pressure (PASP) ≥40 mmHg on ECHO.^[6]^ The gold standard for the diagnosis of PAH is RHC. However, RHC is a highly invasive procedure that is infrequently performed in our setting due to resource constraints. Therefore, to ensure the safety of participants and to preserve resources, RHC was not performed.

This study was approved by the Human Research Ethics Committee of the University of the Witwatersrand (ref. no. M180275). All participants provided written informed consent.

**Fig. 1:**
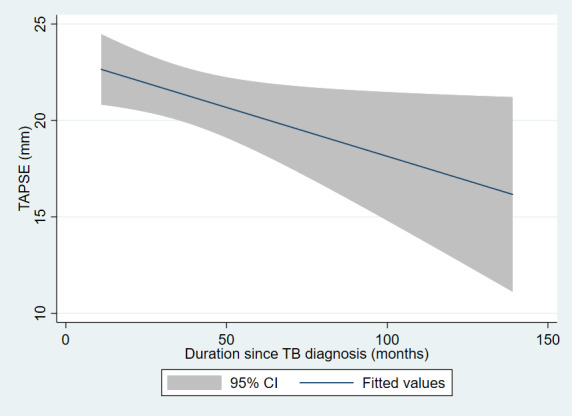
Linear prediction plot showing association between duration since TB diagnosis and tricuspid annular plane systolic excursion (TAPSE). (CI = confidence interval; TB = tuberculosis.)

Frequencies, median and interquartile range were used to describe demographic characteristics, clinical features and investigations (Tables 1 and 2). We explored pairwise associations between numeric variables to determine correlation with each other and with the outcomes of the tricuspid annular plane systolic excursion (TAPSE) and PASP. We plotted linear fit prediction plots with confidence intervals to further explore statistically significant pairwise associations of importance (Fig. 1). Statistical analysis was performed using the SPSS software, version 12 (SPSS Inc., USA).

## Results

We recruited and enrolled 22 participants who were previously treated for smearpositive TB into the study. However, we excluded two participants who had severe bronchiectasis. The median age of the remaining 20 participants was 33 years old (interquartile range (IQR) 29 - 41.5). The majority of the participants (60%) were female and black African (Table 1). All participants had microbiologically confirmed TB at diagnosis, with 80% (n=16) by sputum, 15% (n=3) by bronchial washings and 5% (n=1) by computed tomography (CT) guided fine needle aspirate (FNA) of a lung nodule. The median duration after initial TB diagnosis was 30 months (IQR 14 - 42). No participants had a past or current history of smoking tobacco.

A few participants (15%; n=3) complained of a dry persistent non-productive cough and no complaints of dyspnoea, sputum expectoration or haemoptysis were reported.

Pulmonary function tests showed a median forced expiratory volume in one second: forced vital capacity (FEV1:FVC) ratio of 83% (IQR 73 - 87). We also found that in 15% (n=3) of the participants, the DLCO was reduced after an average of 23 months following diagnosis of TB. All the participants had room air saturation measurements that were above 95% by pulse oximetry (Table 2). More than one-third of the participants (35%; n=7) had one or more features of PH on ECG while the majority of the participants (85%; n=17) demonstrated at least one CXR feature suggestive of PH (Table 2). The majority of the patients (60%) had some degree of bronchovascular distortion and pleuro-parenchymal bands on CXR and the rest had no radiological complications of TB (Table 2). We found no participants that displayed ECHO criteria supportive of PH based on PASP at a median of 30 months after TB diagnosis (Table 3). However, the overall trend of TAPSE values was observed to decline over time after the initial TB diagnosis (Table 2).

## Discussion

In this pilot study, we examined PAH in HIV-negative participants that were previously diagnosed with TB. We found CXR and ECG changes that were suggestive of PAH in some participants after a median duration of 30 months after TB diagnosis. However, no ECHO features of PAH were noted in all participants. A decline in TAPSE was observed over time after TB diagnosis, suggesting a possible decline in right ventricular function.

CXR is readily used as a screening tool for PAH, particularly in low-resource settings and is also able to assess lung parenchymal changes.^[16]^ CXR has a high sensitivity (96.9%) and specificity (99.1%) for detection of PAH when pre-test probability is 50% or more.^[17]^ Miniati *et al*.^[17]^ suggested that CXR findings that are suggestive of PAH are sufficient to warrant further investigation by RHC and that a normal CXR does not exclude the presence of PH. The severity of PH, however, cannot be correlated with CXR changes.^[16]^ We showed in this study that 85% of the participants had CXR features that were suggestive of PH. Although the pre-test probability in these patients was low, these findings still indicated a possible need for further investigations, which would include more definitive investigations such as chest computed tomography (CT) scan and RHC for the confirmation of PAH. This would offer this subgroup of patients novel therapies for PAH under a trial scenario to observe outcomes.

ECG is also another cost-effective screening tool that is not sufficiently sensitive or specific for the diagnosis of PH. For instance, a previous study showed that ECG can be used to detect right ventricular hypertrophy and right-axis deviation in patients with PAH 87% and 79% of the time, respectively.^[18]^ Another study conducted by Al-Naamani et al.^[17]^ demonstrated a positive predictive value that was >80% in specific ECG criteria, namely R/S amplitude in V1 >1 and right-axis deviation of QRS axis >1100.^[19]^ The absence of ECG features does not exclude the presence of PAH.^[18]^ Our study found right-axis deviation in one participant and a dominant R wave in V1.

Transthoracic ECHO is used as a screening tool for suspected PH in individuals with suggestive signs and symptoms. It determines the probability of PH and has a sensitivity of 83%;^[20]^ however, detection of mild PH is limited.^[21]^ PASP is estimated from the peak tricuspid regurgitant jet velocity and the right ventricular size and function is measured by TAPSE.^[22]^ The accuracy of transthoracic ECHO in estimating PASP has been questioned as it may frequently underestimate PASP.^[23]^ This has been attributed to inaccuracies in the estimation of right atrial pressure and poor Doppler imaging of the trans-tricuspid regurgitant jet.^[23]^

The measurement of the tricuspid regurgitant velocity (TRV) by echocardiography improves the probability of detecting PAH. An elevated TRV (>3.4 m/s) suggests a high probability of PAH. We did not include TRV in the analysis for this study, and this is a potential limitation of the study.

The trend of a decreasing TAPSE over time observed in our study highlighted the potential development of PAH over time from initial TB diagnosis. Perhaps the lack of ECHO findings that were suggestive of PAH in this study was due to PAH developing later than the median time of 30 months (IQR 14 - 42). A study undertaken by Humbert *et al*.^[24]^ found a 27-month delay between onset of symptoms and diagnosis of PAH, with 75% of patients having New York Heart Association functional class III at time of diagnosis.^[24]^ This suggested that these individuals may need further prospective follow-up of RV function as well as the performance of more detailed ECHO measures such as TRV to determine if PAH is present.

CT is becoming more accepted as an initial test in the evaluation of PH.[16,25]^[16,25]^ NB: be sure to change rid A main pulmonary artery diameter (PAD) ≥29 mm has an 87% sensitivity in the diagnosis of PH.^[21]^ The size of the pulmonary artery measured on CT is positively correlated with the severity of PH.^[26]^ A mean PAD (mPAD) and mPAD: ascending aorta diameter (AAD) ratio >1 has been shown to have a high correlation (r=0.51 and r=0.53, respectively; p<0.001) with PAP. When an increased mPAD is accompanied by a segmental artery-to-bronchus ratio that is >1:1 in 2 or 4 pulmonary lobes, sensitivity in the diagnosis of PH is 100%.^[26]^ A mPAD:AAD ratio >1 has a sensitivity of 70.8% and a specificity of 76.5% for the diagnosis of PH. The positive predictive value of mPAD: AAD ratio >1 for the diagnosis of PH is 96%.^[27]^ CT is further useful in identifying other causes of PH such as pulmonary vasculature, lung parenchyma and cardiovascular structures.^[28]^

One of the major limitations of this pilot study was the small number of participants that were recruited and enrolled in the study. Identifying participants who were previously treated for TB and had no underlying risk factors for the development of PAH is difficult in a quaternary hospital where most patients suffer from several co-existing pathologies. A more detailed ECHO evaluation of PAH combined with CT may be of value in future studies. Finally, the duration from time of TB diagnosis to enrolment may have been too short to identify the occurrence of PAH. Therefore, this period should be extended in future studies to ensure that sufficient time is provided for PAH to develop.

## Conclusion

Although we did not determine ECHO findings that were suggestive of PAH in the HIV-negative participants that were previously treated for TB, we did find the presence of CXR and ECG features which were suggestive of PH and the possible presence of PAH. This provides enough evidence to prompt further studies with larger sample sizes, a more heterogeneous post-TB population and inclusion of more indepth ECHO analysis to evaluate RV function in combination with radiological studies such as CT to examine the occurrence of PAH after TB.

## Figures and Tables

**Table:** Table 1. Characteristics of patients with previous TB (*N*=20)

Patient characteristics	Mean (SD)*	*n* (%)
Demographic characteristics		
Overall age in years	36.65 (12.83)	
Median (IQR)	33 (29 - 41.5)	
Self-reported race		
Asian		1 (5)
Black		12 (60)
Coloured		1 (5)
Indian		5 (25)
White		1 (5)
Gender		
Male		8 (40)
Female		12 (60)
Clinical features		
Details regarding prior TB infection		
Duration since TB diagnosis (months)	35.65 (31.02)	
Median (IQR)	30 (14 - 42)	
Mode of TB diagnosis		
Sputum		16 (80)
Bronchial washings		3 (15)
CT-guided FNA lung nodule (CXR suggestive of active TB)		1 (5)
Current presence of respiratory symptoms		
Cough		3 (15)
Dyspnoea		0
Sputum expectoration		0
Haemoptysis		0
Comorbidities and risk factors		
Smoking		0
Hypertension		1 (5)
Diabetes		2 (10)
Malignancy		0
Screening for sleep-disordered breathing		
Neck circumference >40 cm		0
Overweight (BMI 25 - 30)		1 (5)
Obesity (BMI >30)		0
Snoring		0
Age >55		2 (5)
Male sex		8 (40)

SD = standard deviation

IQR = interquartile range

FNA = fine-needle aspiration

CXR = chest X-ray

BMI = body mass index

* Unless otherwise specified

**Table:** Table 2. Clinical investigations

Findings on ECG	Median (IQR)	*n* (%)
P		0
Right-axis deviation		1 (5)
S wave in standard lead 1		3 (15)
Q wave in standard lead 3		2 (10)
T wave in standard lead 3		1 (5)
R wave in ventricular lead 1		1 (5)
RVH		0
RV strain		1 (5)
RBBB		0
Findings on chest X-ray		
Elevated cardiac apex		10 (50)
Enlarged right atrium		10 (50)
Enlarged pulmonary arteries		15 (75)
Pruning of peripheral pulmonary vessels		5 (25)
Pleuro-parenchymal bands		12 (60)
Volume loss		2 (10)
Tracheal deviation		0
Spirometry		
FEV_1_	2.77 (2.29 - 3.31)	
FVC	3.41 (2.82 - 3.96)	
Ratio	82.85 (73.10 - 86.85)	
DLCO (%Pred)	99.5 (84.5 - 108.5)	
Low DLCO		3 (15)
Room air saturation	96 (95.5 - 97.0)	
Echocardiography		
LVIDd (mm)	43.5 (41.5 - 48.5)	
LVIDs (mm)	28 (27.5 - 30)	
LVEF (%)	60 (56 - 64)	
RWMA		0
Left atrium (mm)	27.5 (23 - 31)	
Ascending aorta (mm)	23 (21 - 26.5)	
E/a	1.24 (1 - 1.50)	
E/e	6 (5.19 - 8.30)	
Diastolic dysfunction		3 (15)
Aortic regurgitation		1 (5)
Aortic stenosis		0
Mitral regurgitation		2 (10)
Mitral stenosis		0
Tricuspid regurgitation		5 (25)
TAPSE (mm)	21 (19 - 23)	
TAPSE <16 mm		0
PASP (mmHg)	18 (8.5 - 24.5)	
RAP (mmHg)	5 (3 - 9.5)	
IVC (mm)	14 (13 - 18)	
NT-proBNP	26 (16 - 66)	

ECG = electrocardiography

IQR = interquartile range

P = pulmonale

R = right-axis deviation

RVH = right ventricular hypertrophy

RV = right ventricular

RBBB = right bundle branch block

FEV1 = forced expiratory volume in one second

FVC = forced vital capacity

DLCO = diffusing capacity of lung for carbon monoxide

LVIDd = left ventricular internal diameter end diastole

LVIDs = left ventricular internal diameter end systole

LVEF = left ventricular ejection fraction

RWMA = regional wall motion abnormality

TAPSE = tricuspid annular plane systolic excursion

PASP = pulmonary hypertension echocardiography

RAP = right arterial pressure

IVC = inferior vena cava

NT-proBNP = N-terminal pro-B-type natriuretic peptide

**Table:** Table 3. Radiological features of the lung scarring chest X-ray

	*n* (%)
Right lung	
Right upper lobe/zone	10 (50)
Right mid-zone	1 (5)
Right lower lobe/zone	4 (20)
Left lung	
Left upper lobe/zone	1 (5)
Left mid-zone	1 (5)
Left lower zone	1 (5)
Diffuse fibro-cavitary changes	1 (5)
No fibro-cavitary changes	8 (40)
Fibro-cavitary changes limited to one lobe	6 (30)
Fibro-cavitary changes in two lobes	3 (15)
Fibro-cavitary changes in three lobes	2 (10)
